# NTRK2 activation cooperates with PTEN deficiency in T-ALL through activation of both the PI3K–AKT and JAK–STAT3 pathways

**DOI:** 10.1038/celldisc.2016.30

**Published:** 2016-09-20

**Authors:** Haluk Yuzugullu, Thanh Von, Lauren M Thorpe, Sarah R Walker, Thomas M Roberts, David A Frank, Jean J Zhao

**Affiliations:** 1 Department of Cancer Biology, Dana Farber Cancer Institute, Boston, MA, USA; 2 Department of Biological Chemistry and Molecular Pharmacology, Harvard Medical School, Boston, MA, USA; 3 Department of Medical Oncology, Dana Farber Cancer Institute, Boston, MA, USA; 4 Department of Medicine, Brigham and Women's Hospital and Harvard Medical School, Boston, MA, USA

**Keywords:** NTRK2, PI3K, PTEN, T-ALL, STAT-3, targeted therapy

## Abstract

Loss of PTEN, a negative regulator of the phosphoinositide 3-kinase signaling pathway, is a frequent event in T-cell acute lymphoblastic leukemia, suggesting the importance of phosphoinositide 3-kinase activity in this disease. Indeed, hyperactivation of the phosphoinositide 3-kinase pathway is associated with the disease aggressiveness, poor prognosis and resistance to current therapies. To identify a molecular pathway capable of cooperating with PTEN deficiency to drive oncogenic transformation of leukocytes, we performed an unbiased transformation screen with a library of tyrosine kinases. We found that activation of NTRK2 is able to confer a full growth phenotype of Ba/F3 cells in an IL3-independent manner in the PTEN-null setting. NTRK2 activation cooperates with PTEN deficiency through engaging both phosphoinositide3-kinase/AKT and JAK/STAT3 pathway activation in leukocytes. Notably, pharmacological inhibition demonstrated that p110α and p110δ are the major isoforms mediating the phosphoinositide 3-kinase/AKT signaling driven by NTRK2 activation in PTEN-deficient leukemia cells. Furthermore, combined inhibition of phosphoinositide 3-kinase and STAT3 significantly suppressed proliferation of PTEN-mutant T-cell acute lymphoblastic leukemia both in culture and in mouse xenografts. Together, our data suggest that a unique conjunction of PTEN deficiency and NTRK2 activation in T-cell acute lymphoblastic leukemia, and combined pharmacologic inhibition of phosphoinositide 3-kinase and STAT3 signaling may serve as an effective and durable therapeutic strategy for T-cell acute lymphoblastic leukemia.

## Introduction

The class-I phosphoinositide 3-kinase (PI3K) signaling cascade is a central pathway activated in many cancers, including hematological malignancies [1]. Activation of the pathway begins when a receptor tyrosine kinase (RTK) at cell surface is activated, resulting in recruitment of PI3K to the plasma membrane where it phosphorylates phosphatidylinositol (4,5)-bisphosphate (PIP2) to form phosphatidylinositol (3,4,5)-triphosphate (PIP3). This lipid product of PI3Ks acts as a second messenger to trigger diverse signaling cascades that promote cell survival, proliferation and differentiation. Class-I PI3Ks comprise a family of four catalytic isoforms, p110α, p110β, p110δ and p110γ. While p110α and p110β isoforms are ubiquitously expressed, the expression of p110δ and p110γ is largely restricted to leukocytes.

The main negative regulator of this pathway is PTEN, a major tumor suppressor, which dephosphorylates the 3′ position of PIP3 to PIP2 and therefore directly antagonizes the activity of PI3K. The balance between PI3K and PTEN is critical for normal control of cellular activities. Loss of PTEN leads to a constitutively hyperactivated PI3K pathway, an event frequently found in human cancer, including hematological malignancies. For example, anywhere between 8 and 63% of T-cell acute lymphoblastic leukemia (T-ALL) patients have mutated or lost PTEN tumor suppressor gene [2–5], suggesting the importance of the PI3K pathway in T-cell leukemia and providing the rationale for the development of PI3K-targeted therapies in T-ALL.

However, most PI3K inhibitors in early clinical trials are pan-PI3K drugs targeting all class-I PI3K isoforms and have shown modest antitumor activities as single agents [1]. The most effective single-agent PI3K-based therapy to date is Idelalisib (Cal101, GS-1101), a PI3K-p110δ isoform inhibitor. Idelalisib has shown great clinical efficacy in patients with chronic lymphocytic leukemia and indolent lymphoma [6] and has recently been approved by the US Food and Drug Administration for the treatment of these cancers. A recent preclinical study showed that, while p110δ is important for PTEN-deficient T-ALL, p110δ-specific inhibition was not sufficient to suppress tumorigenesis, thus combined inhibition of p110δ and p110γ is required to block T-ALL [7]. Clinical efficacy of inhibiting PI3K in T-ALL has not been reported. Moreover, targeted therapy always faces *de novo* or acquired drug resistance. Given the complexity of cancer genetics and oncogenic signaling networks, combination therapies targeting interdependent signaling events will almost certainly be necessary to achieve effective and durable responses.

In order to identify a molecular target or pathway that cooperates or synergizes with PTEN deficiency to confer oncogenic transformation of leukocytes, we have recently generated a collection of activated RTKs. Since the interleukin (IL3)-dependent Ba/F3 cell model has been widely used for assessing oncogenic kinase signaling and small-molecule kinase inhibitors, we employed this system to screen for RTKs capable of cooperating with loss of PTEN to confer a full growth phenotype of Ba/F3 cells in IL3-independent manner. We found that NTRK2 activation synergizes with PTEN deficiency by engaging both the PI3K/AKT and JAK–STAT3 pathways in T-ALL. We also identified p110α and p110δ as the major isoforms mediating the PI3K/AKT signaling driven by NTRK2 activation and PTEN deficiency in leukocytes. Finally, we demonstrated that a combination treatment with inhibitors targeting PI3K/AKT and JAK–STAT3 achieves effective and durable response both *in vitro* and *in vivo*.

## Results

### NTRK2 activation cooperates with PTEN deficiency to promote the proliferation of Ba/F3 cells in the absence of IL3

**Figure 1:**
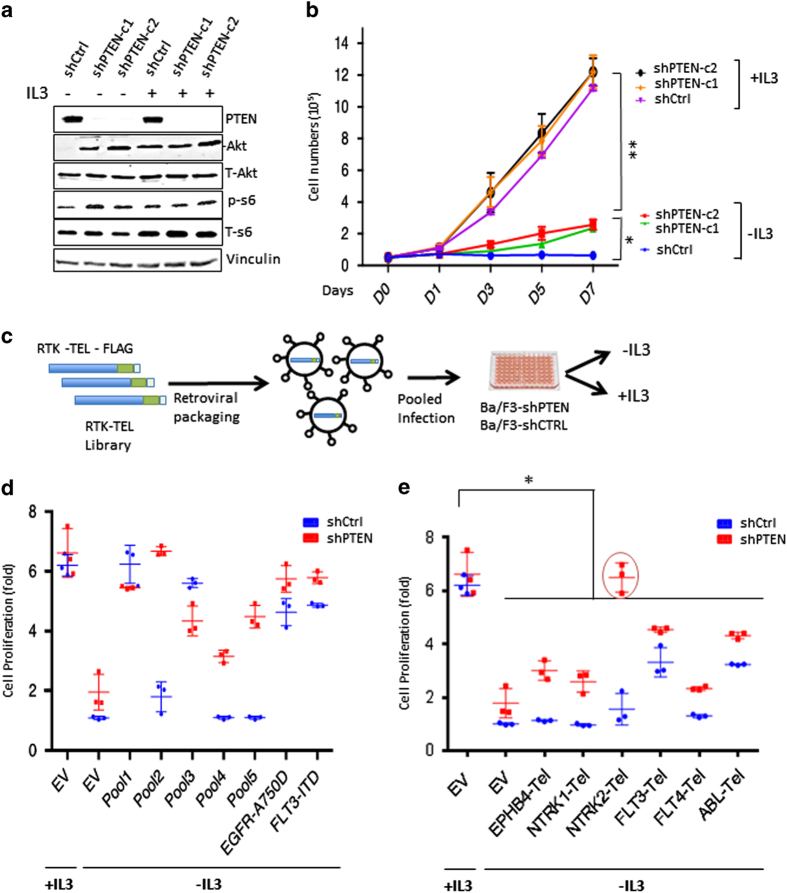
RTK-Tel Library screen identifies NTRK2 as a vulnerability for PTEN-deficient cells. (**a**) Immunoblot analysis of PI3K proteins after PTEN knockdown. Activation of PI3K/mTOR pathways is shown with increased p-Akt 473 in the absence of IL3. (**b**) PTEN knockdown leads to IL3-independent growth. PTEN shRNA expressing Ba/F3 cells were grown both in the absence or presence of IL3 and cells were counted every other day for 7 days (*n*=3). (**c**) Schematic of pool generation. Pools of retroviral vectors encoding 39 human TEL-receptor tyrosine kinases (5 pools of 8 kinases) and positive controls (FLT3-ITD and mut-EGFR-A750del) were transduced into Ba/F3 cells expressing ctrl-shRNA or PTEN shRNA. After selection with neomycin, stable cells that could grow in the absence of IL3 were harvested, and genomic DNA was extracted and sequenced. (**d**) The relative growth of each pool (Pools: Pool 1–5) and positive control cells in the absence or presence of IL3. Relative cell growth was calculated compared with shCtrl mock-infected cells (EV) (*n*=3). Growth obtained over fourfold taken as significant at day 3. (**e**) Candidate RTK-Tels were infected individually to confirm screening results as in **d**.

To test whether PTEN-depletion allows Ba/F3 cells to grow in culture medium lacking IL3, we engineered Ba/F3 cells stably expressing small hairpin RNAs targeting PTEN (shPTEN). Immunoblotting experiments confirmed that PTEN expression was significantly reduced (Figure 1a), and that there was a concomitant increase in AKT and S6 protein phosphorylation without altered expressions of PI3K isoforms (Figure 1a and Supplementary Figure S1A). However, while shPTEN-expressing Ba/F3 cells display an increased growth rate as compared with the control cells in the absence of IL3, these cells grow significantly slower than cells cultured in the presence of IL3 (Figure 1b), suggesting that PTEN deficiency alone is not sufficient to fully transform Ba/F3 cells.

Given the close connection of RTKs and PTEN in signaling regulation, we set out to identify potential receptors that when activated are capable of cooperating with PTEN deficiency to elicit a fully transformed phenotype in Ba/F3 cells. We generated a library in a retroviral expression vector (pWZL-Neo) harboring RTKs consisting of 39 unique human RTKs activated by C-termini fusion to the TEL dimerization domain (RTK-Tel) (Figure 1c and Supplementary Table S1). The library was screened in Ba/F3 cells harboring shPTEN as five sub-pools each consisting of eight activated kinases (Figure 1c and d). Fetal liver kinase-internal tandem duplication (FLT3-ITD) and EGFR-E746-A750del, both of which have previously been shown to confer IL3-independent growth of Ba/F3 cells [8, 9], were included as positive controls in the screen (Figure 1d). As expected, in the absence of IL3, shCtrl cells infected with the empty vector failed to grow, and mock-infected shPTEN cells grew modestly, while the same cells grew well in the presence of IL3 (Figure 1d). Cells expressing FLT3-ITD and the mutant EGFR-A750del grew robustly in the absence of IL3 regardless of PTEN status (Figure 1d). While pools 1 and 3 fostered robust proliferation of both shCtrl and shPTEN Ba/F3 cells in the absence IL3, pools 2, 4 and 5 allowed shPTEN cells, but not shCtrl cells to grow without IL3 (Figure 1d). These results suggest that transformation by some of the TEL-RTK fusions requires concomitant loss of PTEN expression.

Next, we extracted genomic DNA from these shPTEN-expressing cells, performed PCR analyses with vector-specific primers followed by sequencing to identify kinases enriched in our screen. In total, 6 Tel-RTKs out of the total of 39 Tel-RTKs were scored: Ephrin type-B receptor 4 from pool 1, Neurotrophic Tyrosine Receptor Kinase Type 1 (NTRK1) and Neurotrophic Tyrosine Receptor Kinase Type 2 (NTRK2) from pool 2, FLT3 from pool 3, FLT4 from pool 4 and ABL from pool 5 were found to be enriched for their expressions in these cells (Supplementary Table S1). We further evaluated these six scored kinases by introducing them individually into shCtrl- or shPTEN-expressing Ba/F3 cells. FLT3-Tel and ABL-Tel were able to promote Ba/F3 cells grow in the absence of IL3 regardless of the PTEN status (Figure 1e and Supplementary Figure S1B), consistent with previous studies that both kinases have transforming activities [10, 11]. The three RTKs, NTRK1 (also known as TrkA), Ephrin type-B receptor 4 and FLT4 displayed modest transforming activities in Ba/F3 cells when Pten is deficient (Figure 1e). Notably, only NTRK2 (also known as TrkB) robustly synergized with PTEN deficiency to promote a robust proliferation in the absence of IL3 that is comparable or higher than the growth rate of cells in the presence of IL3 (Figure 1e and Supplementary Figure S1C). Moreover, we show that overexpression of a full-length NTRK2 also led to cytokine-independent growth similar to what was observed with Tel-NTRK2 (Supplementary Figure S1D and E), indicating that NTRK2 activation leads to transformation of Ba/F3 cells in the absence of PTEN.

### NTRK2 overexpression is enriched in a subset of PTEN-deficient T-ALL

**Figure 2:**
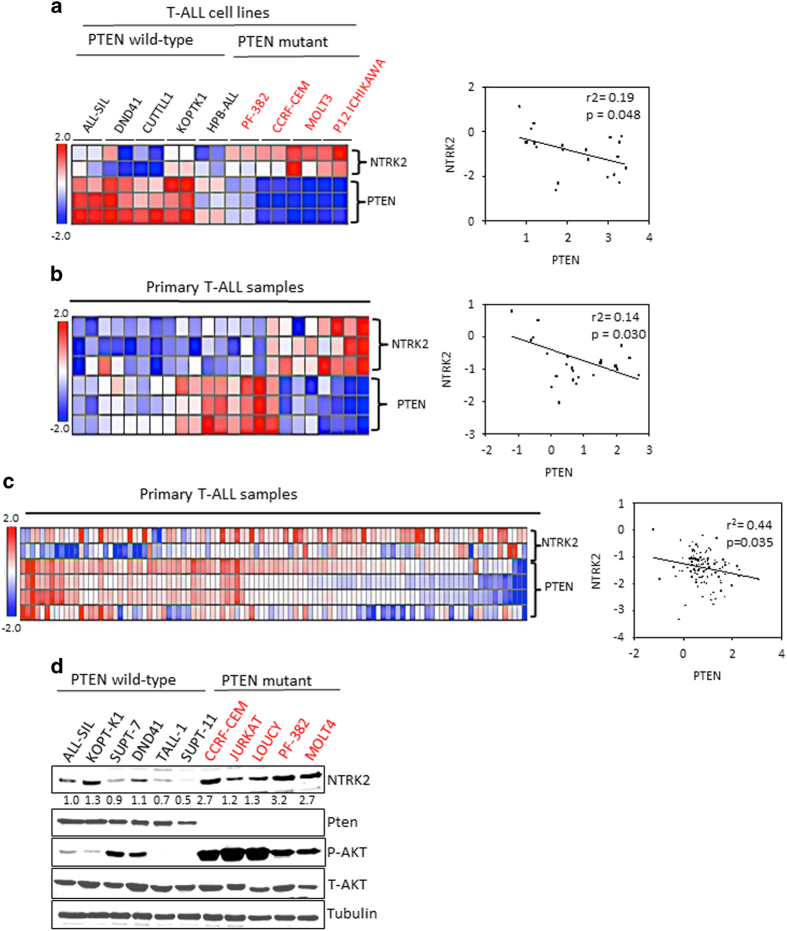
Correlation of PTEN deficiency with NTRK2 expression in T-ALL cells. (**a**) Heatmap showing inverse correlation of PTEN transcripts level with NTRK2 gene in T-ALL cell lines. Samples in the indicated data sets were grouped into PTEN wild-type or mutant PTEN gene signature [4] (CCRF-CEM cells have frameshift at aa 28, PF382 cells frameshift at aa 84, RPMI8402 has frameshift at aa 236 and P12-ICHIKAWA cells has premature stop codon at aa 267). NTRK2 expression inversely correlates with that of PTEN. Linear regression was determined using GraphPad Prism. The linear regression Pearson's correlation coefficient (*R*^2^) and its *P-*value are indicated. (**b**, **c**) NTRK2 gene expression is inversely correlated with PTEN expression in two independent cohorts, Dik *et al.* [12], *n*–23 and Soulier *et al.* [13], *n*=104, of primary T-ALL. (**d**) Immunoblot analysis of NTRK2 protein expression in T-ALL cell lines. NTRK2 protein expression in PTEN wild type or mutant T-ALL cell lines. Tubulin was used as a loading control.

To assess the clinical relevance of NTRK2 activation and PTEN, we analyzed gene expression data [4] from a panel of T-ALL cell lines in the Oncomine Database (Oncomine), and found that the levels of the NTRK2 transcript were consistently higher in PTEN-deficient T-ALL cell lines compared with PTEN wild-type cells (Figure 2a). Moreover, NTRK2 expression was inversely correlated with PTEN expression in subsets of primary T-ALL patients as demonstrated by two independent data sets [12, 13] (Figure 2b and c). We performed western blotting analysis of a panel of T-ALL cell lines and found that most PTEN mutant T-ALL cell lines have more abundant NTRK2 compared with cells with wild-type PTEN (Figure 2d). We also found that levels of phospho-AKT (Ser473) are inversely correlated with PTEN expression as expected (Figure 2d). Together, these observations indicate that NTRK2 overexpression may be enriched in a subset of PTEN-deficient T-ALL.

### PTEN deficiency and NTRK2 activation cooperate to increase PI3K/mTOR and JAK–STAT3 signaling

**Figure 3:**
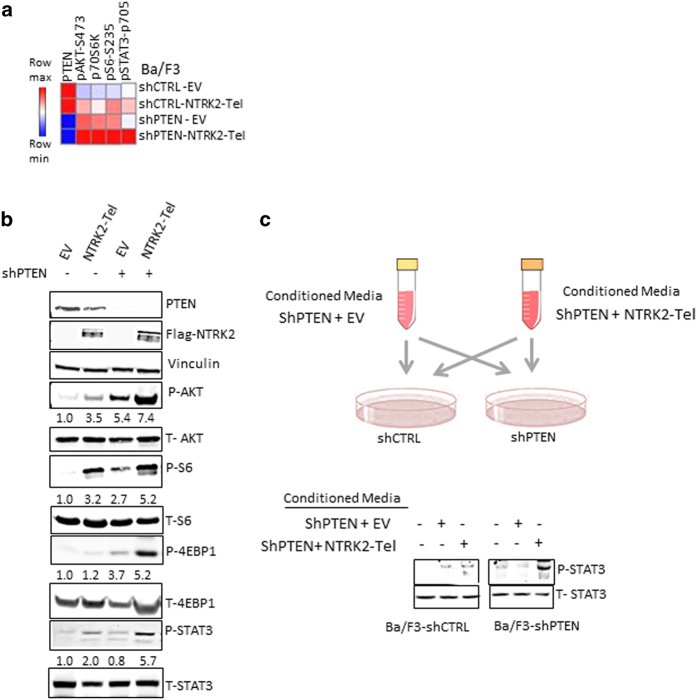
The cooperative effect of PTEN loss and NTRK2 activation on PI3K/AKT and STAT3 signaling pathways. (**a**) Heatmap of increased PI3K/mTOR and STAT3 activation generated by RPPA assay in Ba/F3 cells. Cells from Ba/F3-shCTRL-EV, shCTRL-NTRK2-Tel, shPTEN-EV and shPTEN-NTRK2-Tel were lysed and subjected to reverse phase protein array assay. (**b**) Immunoblotting analysis of AKT and STAT3 signaling in the Ba/F3 cells as indicated. Quantification of P-AKT and P-STAT3 was normalized to total AKT or total STAT3 levels. (**c**) Immunoblotting analysis of Y705 pSTAT3 in Ba/F3-shCTRL and shPTEN cells cultured for 2 h in conditioned medium from Ba/F3-shPTEN or Ba/F3-shPTEN-NTRK2-Tel-overexpressing cells for 24 h.

To assess the signaling changes upon NTRK2 activation in the PTEN-deficient setting, we performed quantitative reverse phase protein array assay (RPPA) [14] in Ba/F3 cells expressing shPTEN and NTRK2-Tel. RPPA assay detected activation of the PI3K/mTOR pathway as indicated by increased p-Akt and p-S6 levels upon PTEN knockdown, while overexpression of NTRK2 led to activation of the STAT3 signaling pathway in addition to Akt phosphorylation (Figure 3a). When combined, these two events synergized in the activation of PI3K/mTOR and STAT3 signaling, as shown by markedly increased levels of p-Akt, p-S6, p-4EBP1 as well as STAT3 activation (Figure 3a). Consistent with this finding, immunoblotting analysis also showed further enhancement of PI3K/mTOR pathway activity and STAT3 signaling in cells with concomitant PTEN loss and NTRK2 activation compared with either event alone (Figure 3b). These results suggest that loss of PTEN and activation of NTRK2 cooperate to increase these two signaling pathways, PI3K/mTOR and JAK–STAT3, known to be critical for the transformation of leukocytes [15].

To further understand how STAT3 is phosphorylated upon NTRK2 activation, we cultured Ba/F3-shCtrl and shPten cells with conditioned medium from BA/F3 ShPTEN +EV or Ba/F3 ShPTEN-NTRK2-Tel cells (Figure 3c). Notably, Ba/F3-ShPTEN cells cultured with conditioned medium harvested from Ba/F3 ShPTEN-NTRK2-Tel cells showed markedly increased STAT3 phosphorylation as compared with any other conditions (Figure 3c). These results suggested that NTRK2 activation led to increased STAT signaling via an autocrine loop.

### p110α and p110δ isoforms of PI3K mediate PI3K/AKT/mTOR signaling in NTRK2 overexpression and PTEN-deficient T-ALL cells

**Figure 4:**
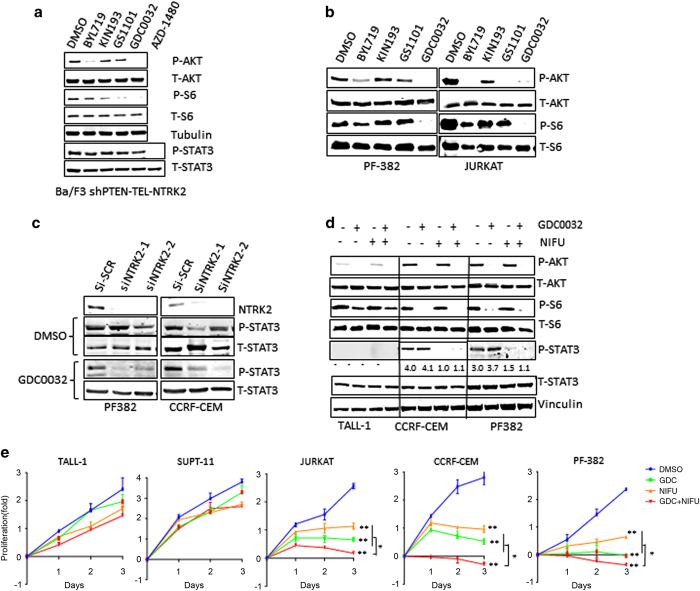
Effects of combined inhibition of PI3K and STAT3 pathways on PTEN-mutated T-ALL cells. (**a**) Short-term treatment of Ba/F3-shPTEN-NTRK2-Tel cells with isoform-selective inhibitors and Pan-PI3K inhibitor GDC-0032. Cells were treated with DMSO, BYL719, KIN193, GS-1101, GDC-0032 (1 μm) or JAK–STAT inhibitor AZD-1480 (1 μm) for 3 h (*n*=3) for each treatment. (**b**) Immunoblot analysis of P-Akt and p-S6 in PF382 and JURKAT cells. Cells were treated with the same inhibitors as in **a**. (**c**) Combination of siNTRK2 and GDC0032 leads to reduced STAT3 phosphorylation. Cells were subjected to siNTRK2 treatment for 72 h, following with or without 1 μm GDC-0032 treatment. Cell lysates were prepared for immunoblotting analysis. (**d**) Immunoblotting analysis of P-Akt and p-STAT3 in T-ALL, CCRF-CEM and PF382 cells. Cells were treated with the GDC-0032, nifuroxazide (NIFU, 10 μm) alone or in combination for 3 h. (**e**) Proliferation of T-ALL cells in the presence of GDC-0032 (1 μm) or nifuroxazide (NIFU, 10  μm) or both for 3 days. The graphs indicate means±s.d. for three experiments. *P<0.05, **P<0.01, one-way ANOVA test.

We were curious as to which PI3K isoforms might be responsible for PI3K signaling in leukocytes with PTEN deficiency and NTRK2 activation. Given the essential role for the p110α catalytic subunit of PI3K in transducing oncogenic signals downstream of upregulated or mutated RTKs and oncogenes such as Ras in hematological malignancies [16], it seemed a likely candidate but it was also possible that p110β which is known to be activated by PTEN loss or p110δ might also be contributing [6, 17]. Upon short-term treatments of Ba/F3 ShPTEN-NTRK2-Tel as well as T-ALL cell lines with PI3K isoform-selective inhibitors, we found that BYL719 [18] (a p110α-selective inhibitor) greatly reduced AKT but not S6 phosphorylation, whereas GS-1101 [19] (idelalisib, a p110δ-selective inhibitor) inhibited S6 protein phosphorylation or AKT phosphorylation depending on cell lines (Figure 4a and b). KIN193 (a p110β-selective inhibitor) [20] had little to modest effect on both AKT and S6 phosphorylation (Figure 4a and b). Notably, GDC-0032 [21], a p110β-sparing PI3K inhibitor, completely abolished both Akt and S6 phosphorylation, suggesting that targeting more than one isoform is required for effective inhibition of the PI3K/mTOR pathway (Figure 4a). Not surprisingly, GDC-0032 had little effect on STAT3 phosphorylation in these cells compared with JAK–STAT inhibitor AZD-1480, suggesting that, while the cooperative activity of NTRK2 with PTEN loss leads to STAT3 activation, PI3K inhibition alone was not sufficient to block the STAT3 signal.

### Combined inhibition of PI3K and STAT3 signaling pathways is highly effective in blocking the growth of NTRK2 overexpression and PTEN-deficient T-ALL cells

We next assessed whether knockdown of NTRK2 could lead to reduced STAT3 phosphorylation in T-ALL cells in a PTEN-dependent fashion. Notably, PTEN mutant cell lines, CCRF-CEM and PF382, displayed a much higher phosho-STAT3 (p-STAT3) level than that of PTEN wild-type TALL-1 and Sup-T11 cells (Supplementary Figure S2A). While, siRNA-mediated knockdown of NTRK2 had little effect on the p-STAT3 level, combination of siNTRK2 with GDC-0032 led to markedly reduced STAT3 phosphorylation (Figure 4c), supporting the notion of cooperative roles of NTRK2 activation and PTEN loss on STAT3 activation.

To evaluate the efficacy of combined inhibition of both PI3K/Akt and STAT signaling on these PTEN-deficient cells, we treated CCRF-CEM and PF382 with GDC-0032 and a STAT3 inhibitor (nifuroxazide) [22] alone or in combination. The PTEN-proficient cell line TALL-1 was used as a control for baseline PI3K/mTOR and STAT3 activities (Figure 4d). As expected, GDC-0032 treatment reduced phosphorylation of AKT and S6 protein, while nifuroxazide suppressed phosphorylation of STAT3, and in combination they blocked both PI3K/mTOR and STAT3 signaling pathways in PTEN-deficient cells (Figure 4d). While either GDC-0032 or nifuroxazide alone reduced the proliferation of PTEN-deficient CCRF-CEM, PF382 and Jurkat cells, combined treatment reduced proliferation of these cells to a significantly greater extent than either inhibitor alone (Figure 4e). In contrast, combination treatment had a limited effect on the PTEN-proficient cell lines TALL-1 and SUPT-11 cells (Figure 4e). Moreover, for PF382 and CCRF-CEM cells, we performed a combinatorial matrix experiment in which we treated cells with wide range of GDC-0032/nifuroxazide concentration pairings. Combinatorial inhibition indices of cell viability after combined treatment suggested that combined treatment led to more cell growth suppression in PF382 and CCRF-CEM (combination indices of 0.55, 0.65 where a combination index <1 implies synergy) [23] (Supplementary Figure S2B). These results suggest that combined pharmacologic inhibition of PI3K and STAT signaling could be an effective therapeutic strategy for PTEN-deficient T-ALL.

**Figure 5:**
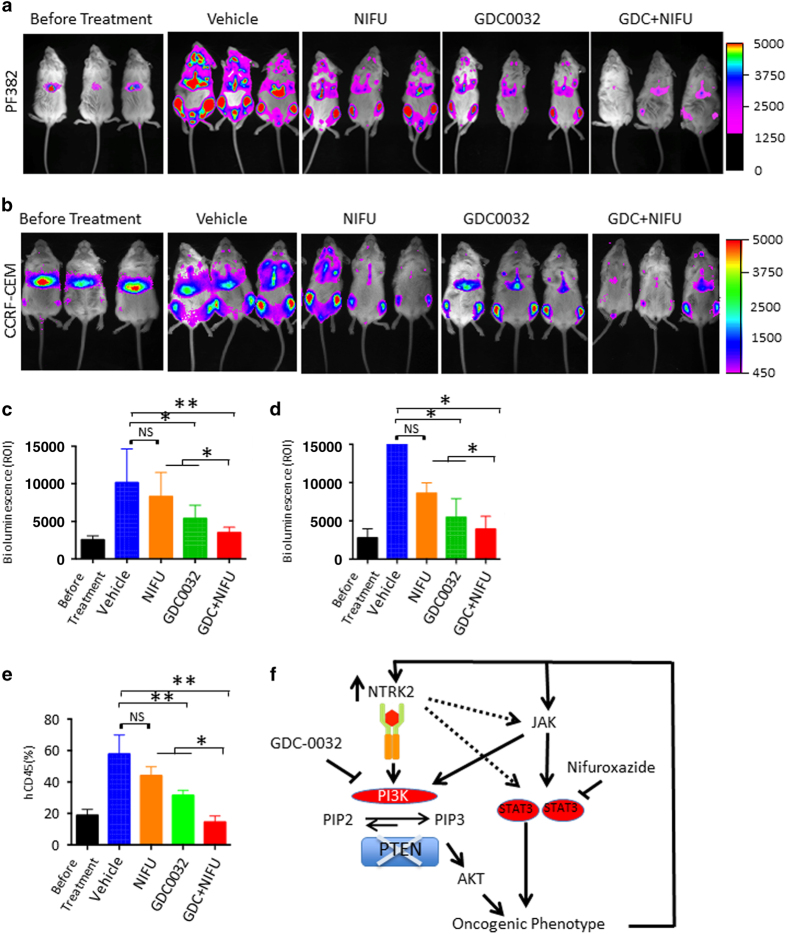
Combined inhibition of PI3K and STAT3 pathways suppressed leukemia in murine xenograft models of T-ALL cell lines. (**a**) Bioluminescence imaging of mice before treatment or 26 days after injection with 3×10^6^ PF382-luciferase cells and treatment once daily with 15 mg kg^−1^ GDC-0032 or 313 mg kg^−1^ nifuroxazide or the combination. The intensity scale of the bioluminescence signal applies to all images. (*n*=4 for each group). (**b**) Bioluminescence imaging of mice before treatment or 26 days after injection with 3×10^6^ CCRF-CEM-luciferase cells and treatment once daily with 15 mg kg^−1^ GDC-0032 or 313 mg kg^−1^ nifuroxazide or the combination. The intensity scale of the bioluminescence signal applies to all images (*n*=5 for each group). (**c**) Quantification of Luciferase signaling from mice injected with PF382 cells (*n*=4 for each group). *P<0.05, **P<0.01, one-way ANOVA. (**d**) Quantification of Luciferase signaling from mice injected with CCRF-CEM cells (*n*=4 for each group). *P<0.05, **P<0.01, one-way ANOVA. (**e**) Quantification of human CD45 positive PF382 cells in peripheral blood of mice from **a** (*n*=4 for each group). *P<0.05, **P<0.01, one-way ANOVA. (**f**) A working model of signaling cascades downstream of NTRK2 and PTEN showing activation of PI3K and STAT3 signaling pathways.

Although GDC-0032 is currently used in clinical trials for solid tumors (NCT02390427) and nifuroxazide is available in the clinic in many countries worldwide and is generally well-tolerated [24], these drugs have not been tested in combination against T-ALL *in vivo*. To test the *in vivo* effect of GDC-0032 and nifuroxazide in T-ALL, we performed xenograft studies in mice. PF382 and CCRF-CEM cells transduced with a luciferase-expressing lentiviral vector were injected intravenously into immunodeficient mice, and drug administration was initiated 5 days after transplantation by daily oral gavage, when the bioluminescence images showed detectable leukemic cells (Figure 5a). All animals were killed 21 days after treatment, when the vehicle-treated animals were lethargic. Bioluminescence images obtained after 21 days of drug treatment showed a significantly reduced disease burden in response to GDC-0032 alone but the greatest suppression of luciferase signal was seen in the combination treatment group compared with the vehicle-treated group (Figure 5a and b). Compared with mice before treatment, quantification of luminescence signal showed that only the mice treated with the combination of agents showed stable disease or partial response (Figure 5a–d). Flow cytometric analysis of human CD45^+^ leukemic cell frequency in peripheral blood compared with murine CD45 confirmed our imaging in showing that combination treatment lead to the highest degree of suppression of T-ALL cells in peripheral blood (Figure 5e and Supplementary Figure S2C). Thus, targeting PI3K with GDC-0032 and STAT3 with nifuroxazide has improved *in vivo* efficacy and suggests a novel combination therapy for T-ALL.

## Discussion

Despite major advances in the treatment of T-ALL, targeting T-ALL cells that are refractory to current therapy remains a major clinical challenge due to a lack of novel targets. In this study, we report that NTRK2, a kinase that emerged from an unbiased activated RTK screen, may be a potential therapeutic target in a subset of T-ALL with PTEN deficiency.

The TRK family of receptors is comprised of three members, NTRK1, NTRK2 and NTRK3, that can be activated by their ligands nerve growth factors, brain-derived neurotrophic factors (BDNF) and neurotrophin 3, respectively [25, 26]. Notably, only NTRK2 was scored as comparable to positive control cells in our screen. NTRK2 has previously been implicated in several types of cancers including neuroblastoma, medulloblastoma, Wilm’s tumor and adenocarcinomas of the lung, prostate and pancreas as well as multiple myeloma [27–30]. NTRK2 has been previously shown to increase metastatic potential by suppressing anoikis [31] and gene fusions involving NTRK2 have been found in pilocytic astrocytoma [28] as well as pontine glioma [27]. In hematopoietic cells, B and T lymphocytes as well as monocytes have been shown to produce BDNF [32]. NTRK2 and its ligand BDNF have been shown to be co-expressed in acute leukemia blasts, and this negatively correlates with survival of leukemia patients [33]. Mice with bone marrow transduced with both NTRK2 and BDNF developed AML and T-ALL [33], suggesting a role of NTRK2 in leukemia pathogenesis. Interestingly, tumors in a murine model of T-ALL induced by TRK receptors activations also acquire mutations in NOTCH1 and loses PTEN during clonal evolution, leading to hyperactivation of mTORC1/2 signaling [34].

TRK receptors have been reported to be expressed in hematopoietic progenitors and implicated to have important roles during hematopoiesis [35]. Maroder *et al.* reported inversely correlated expression of TRKB during T-cells development, being highly expressed in CD4^−^8^−^ immature thymocytes and progressively declining in CD8^+^ and CD4^+^ single-positive and CD4^+^8^+^ more mature thymocytes [36]. How NTRK2 is activated in T-ALL has not yet been studied in detail. However, NTRK2 expression has been found to be induced by HIF-1 transcriptional activator in neuroblastoma cells [37]. Considering that loss of PTEN regulates hypoxia and HIF-1 stability in GBM, further mechanisms underlying aberrant expression of TRKB in PTEN-deficient leukemic cells and HIF-1 needs to be determined [38].

Consistent with these findings, we found that PTEN-deficient T-ALL cells have increased expression of NTRK2 in T-ALL cell lines as well as primary patient samples. Mechanistically, we found that NTRK2 activation leads to increased activation of both PI3K/mTOR and Jak–STAT3 signaling pathways, which are greatly enhanced in the absence of PTEN, providing an explanation for the synergistic cooperation of the two events necessary to fully rescue the growth of Ba/F3 cells in the absence of IL3. Our co-culture experiment with conditioned medium harvested from Ba/F3-shPTEN-NTRK2-Tel-expressing cells indicates that NTRK2 activation may lead to a JAK–STAT3 activation, at least in part, through possible increased production of cytokines that may be further boosted by PTEN loss (Figure 5f). To our knowledge, this is the first study to demonstrate that NTRK2 activation and PTEN deficiency cooperate in an aggressive leukemia.

Our finding that NTRK2 activates PI3K/mTOR signaling is consistent with recent studies that NTRK2 promotes lung cancer and chondrosarcoma survival and metastasis by activating PI3K/AKT signaling [39, 40]. Studies with neurons showed that PI3K does not directly bind to NTRK2 [41, 42]. However, when activated with BDNF ligand, NTRK2 autophosphorylates key tyrosine residues in its juxtamembrane domain. These then act as docking sites for adaptor molecules including SHC1, FRS2, SH2, GRB2 as well as IRS-1 and IRS-2, which regulate key signaling cascades including PI3K [41, 43]. Thus, NTRK2 might activate PI3K by recruiting adaptor proteins. Given that both p110α and p110δ isoforms mediate RTK signaling but p110δ is largely expressed in leukocytes, it is interesting that inhibition of p110α is more effective than inhibition of p110δ in reducing cellular signaling and proliferation. PTEN-deficient T-ALL cells has previously been shown to depend on p110δ and p110γ isofroms [7]. When an activated RTK is also present in PTEN-null cells, the isoform dependency appears to have shifted towards key isoforms mediating RTK signaling, as we reported previously [44, 45]. It is thus not surprising that GDC-0032, a p110β-sparing inhibitor that targets both p110α and p110δ, displays superior activity to single isoform-specific inhibitors. Notably we have found that oncogene of RTK activation can also alter p110 isoform utilization in solid tumors as well [44, 46].

Finally, inhibition of PI3K alone in these cells has little effect on STAT3 phosphorylation, suggesting that Jak–STAT3 activation might engender at least partial resistance to PI3K inhibition. Indeed JAK/STAT positive feedback activation has previously been shown to cause resistance to PI3K resistance in solid tumors [47, 48]. Notably, we demonstrated that combined inhibition of PI3K and STAT3 with GDC-0032 and nifuroxazide in T-ALL cells as well as in xenograft tumor models of T-ALL resulted in maximal suppression of cellular proliferation and tumor progression *in vivo*. Together, our studies suggest that a combination therapy that targets both the PI3K/mTOR and the JAK/Stat pathways may reduce the frequency of treatment failures and decrease the risk of relapse in T-ALL patients. Our combination therapy may also be helpful for the treatment of other malignancies induced by altered RTK signaling coupled with an autocrine cytokine loop.

## Materials and methods

### Cell culture

Ba/F3 cells were obtained from Kira Gritsman (Dana Farber Cancer Institute and Brigham and Women’s Hospital, Boston, MA, USA) and maintained in RPMI with 10% fetal bovine serum (FBS) and IL3 (10 ng ml^−1^) (Peprotech, Rocky Hill, NJ, USA). Hematological cancer cell lines were obtained from Thomas Look (Dana Farber Cancer Institute, Boston, MA, USA) and cultured in RPMI (Life Technologies, Waltham, MA, USA) supplemented with 10% FBS (Life Technologies) and 1% Pen/Strep (Life Technologies). For luciferase-expressing PF382 cells, 8 μg pLENTI-Luciferase-Blasticidin vector (gift from H Chang, Dana Farber Cancer Institute, Boston, MA, USA), 2 μg of the packaging plasmid VSVG and 1 μg of D-8-9 helper plasmid were cotransfected into HEK293T packaging cells with 20 μl Fugene 6 (Promega, Madison, WI, USA). After 72 h, the culture supernatants containing lentivirus were collected and filtered (0.45 μm). Cells were transduced twice with lentivirus at 37 °C overnight. Two days after transduction, cells were selected in 35 μg ml^−1^ blasticidin for 10 days. Polyclonal cells were analyzed and confirmed for luciferase expression using the Promega Luciferase assay system, and then expanded for transplantation.

### Virus production and screening of TEL-RTK library of Ba/F3 cell

Phoenix cells were grown in Dulbecco's modified Eagle's medium containing 10% FBS and 1% Pen/Strep. Ba/F3 cells were cultured in RPMI medium containing 10% FBS and 1% Pen/Strep with IL3. Virus was produced as recently described with small modifications. Two micrograms of each TEL-RTK were combined into a single pool transfected into 90% confluent Phoenix cells cultured in Dulbecco's modified Eagle's medium in a 10 cm^2^ flask using FuGENE6 (Promega) according to the manufacturer’s protocol. After 16 h, media were exchanged with fresh media and 2 days post-transfection, the viral supernatant was harvested and filtered. In one well of a 24-well plate, 1 ml of viral soup was added to 50% confluent Ba/F3 cells contained in 0.5 ml of RPMI media, including IL3, and 0.002 ml of Polybrene. Forty-eight hours after infection, the infected Ba/F3 culture was split and transferred to 1.5 ml of RPMI media containing IL3 (10 ng ml^−1^) and neomycin at a final concentration of 500 μg ml^−1^. After an additional 5 days, the neomycin-selected cells were washed twice with one volume of media lacking IL3 to remove dead cells and trace IL3. The cells were transferred to six-well plates containing 2 ml of IL3-free media and subjected to MTS assay.

### Small-molecule inhibitors

KIN193 (1 μm) and GDC-0032 (1 μm) from Haoyuan Chemexpress (Shanghai, China); NVP-BYL719 (1 μm), GS-1101 (1 μm) and AZD-1480 (1 μm) were synthesized by Chemitek (Indianapolis, IN, USA) were from and nifuroxazide (10 μm) were from MP Biomedicals (Santa Ana, CA, USA).

### Combination matrix experiment

Cells were plated in 96-well plates and treated with different concentrations of GDC-0032 (1, 0.5 and 0.25 μm) and nifuroxazide (10, 5, 2 μm) either alone or in combination pairings and MTS assay was performed after 48 h. The results were put into CompuSyn software (http://www.combosyn.com) and combination indeces were calculated as using the Chou–Talalay method [23].

### Mice studies

About 5–6 weeks age CIAE NOG mice were purchased from Taconic (Salisbury, CT, USA). All mice were housed in a pathogen-free animal facility at Children’s Hospital Boston or at Dana Farber Cancer Institute in sterile conditions. 3×10^6^ millions PF382- and CCRF-CEM Luciferase cells were injected through tail vein to perform bioluminescence imaging. After 5 days, mice were treated either with 15 mg kg^−1^ GDC-0032 dissolved in 0.5% methylcellulose, 0.2% Tween-80 and 313 mg kg^−1^ nifuroxazide dissolved in 0.5% methylcellulose and 0.2% Tween-80.

### Bioluminescence imaging

Anesthetized mice were injected i.p, with ketamine and xylazine, and d-luciferin (Promega) was used to monitor luciferase gene expression *in vivo*. Kodak Image Station 4000MM (KODAK, New Haven, CT, USA) was used to perform imaging and analyzed with KODAK Molecular Imaging Software (KODAK).

### Flow cytometry

About 0.5 ml peripheral blood was harvested and subjected to RBC lysis using RBC lysis solution (Qiagen, Valencia, CA, USA). Cells were stained with the PE-conjugated anti-human CD45 (BD, Franklin Lakes, NJ, USA) and FITC conjugated Mouse CD45.1 antibodies and then analyzed on BD FACSCanto II instruments.

### Western blotting

Whole-cell protein lysates were prepared from single-cell suspensions of cell lines. Western blotting was performed with the following modifications. Blocking was performed in 1× TBS, 5% nonfat milk. Primary antibodies used were as follows: PTEN (Cell Signaling, Danvers, MA, USA, #9559) p110α (Cell Signaling #4249), p110β (Cell Signaling #3011), AKT (Cell Signaling #9272), phospho-AKT (Ser473) (Cell Signaling #4060), S6 (Cell Signaling #2217), phospho-S6 (Cell Signaling #2215), Phospho-4EBP1 (Cell Signaling #9451), T-4EBP1 (Cell Signaling #9452), p-STAT3 (Cell Signaling #9131) and T-STAT3 (BD #S21320), ERK (Cell Signaling #9102), phospho-ERK (Cell Signaling #9101), α-Vinculin, FLAG (Sigma-Aldrich, St Louis, MO, USA, #M2) and TRKB (Santa Cruz, Dallas, TX, USA, #SC-12). Fluorophore-conjugated secondary antibodies anti-mouse 680 (Invitrogen, Waltham, MA, USA) and anti-rabbit 800 were used. Signal intensity with background correction was quantified using LICOR Image Studio software (Lincoln, NE, USA).

### MTS proliferation assays

Cell lines were plated at 0.3–0.5×10^5^ cells/well in 96-well plates and incubated in drug solution for 72 h. Aqueous One reagent (Promega) was added and then incubated for 1 h at 37 °C, after which absorbance readings at 490 nm were taken with a correction reading of 700 nm.

### Knockdown of NTRK2

SiRNA(s) targeting human NTRK2 were obtained from IDT (Integrated DNA Technologies, Coralville, IA, USA) and the siRNA sequences were siNTRK2-1 ACCACGAACAGAAGUAAU and SiNTRK2-2 CCAAGAAUGAGUAUGGG. SiRNAs were transfected twice in 2 days in six-well plates in CCRF-CEM and PF382 cells using RNAiMax (Invitrogen) transfection reagent in the absence of serum and cultured for 3 days in the absence of serum and lysates cells were prepared as described above.

### RPPA assay

Stable cell lines were pelleted, washed twice with 1× phosphate-buffered saline and lysed with 1% Triton X-100, 50 mm HEPES, pH 7.4, 150 mm NaCl, 1.5 mm MgCl_2_, 1 mm EGTA, 100 mm NaF, 10 mm Na pyrophosphate, 1 mm Na_3_VO_4_, 10% glycerol and protease inhibitor cocktail. Whole-protein lysates were diluted to 1 μg μl^−1^ and sent to MD Anderson RPPA Core facilities at MD Anderson Cancer Center, Department of Systems Biology, Houston, TX, USA.

### Statistics

Graphpad Prism 6.0 (La Jolla, CA, USA) was used for all statistical analyses. One-way Anova statistical test is used in each figure legend. Error bars reflect mean±s.e.m. *P-* values less than 0.05 or 0.01 were considered significant.
