# Supplementary figures and images for: NTRK2 activation cooperates with PTEN deficiency in T-ALL through activation of both the PI3K–AKT and JAK–STAT3 pathways
Source: Cell Discov. 2016 Sep 20;2:16030–. doi: 10.1038/celldisc.2016.30 (PMC5029543; doi:10.1038/celldisc.2016.30)

A

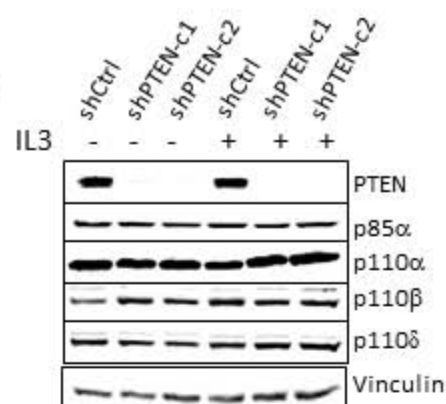

B

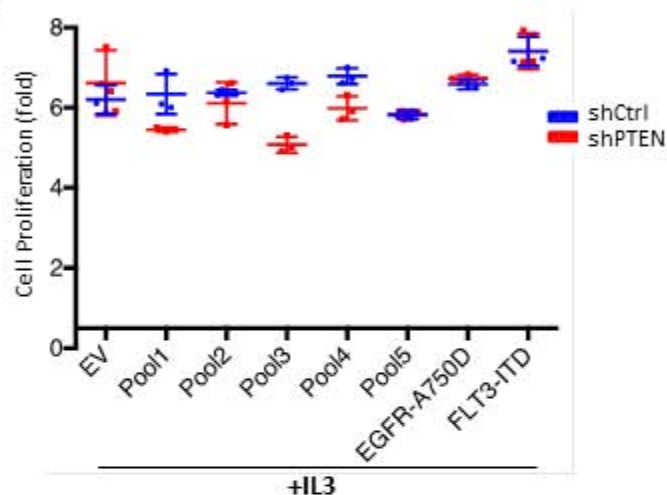

C

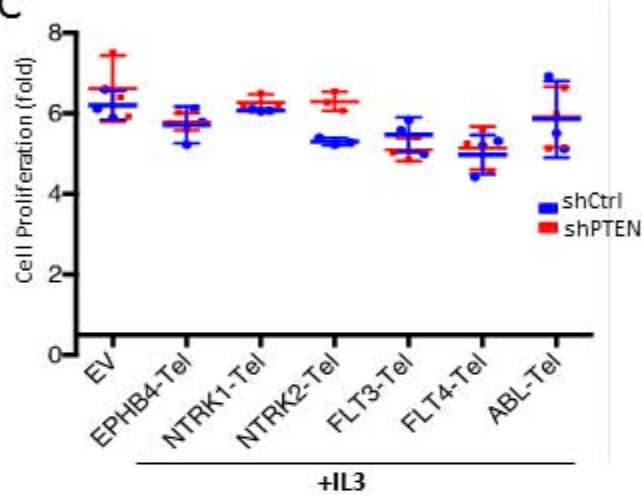

D

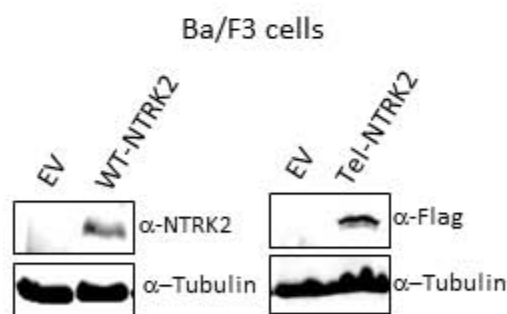

E

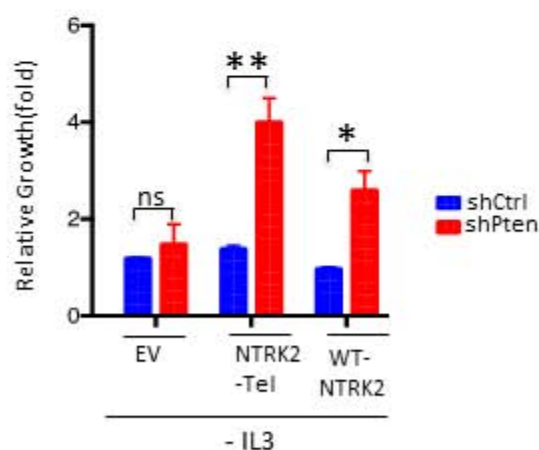

Supplement: Supplementary Figure S1 [file celldisc201630-s1.pdf]

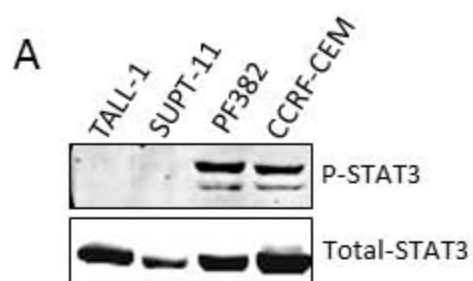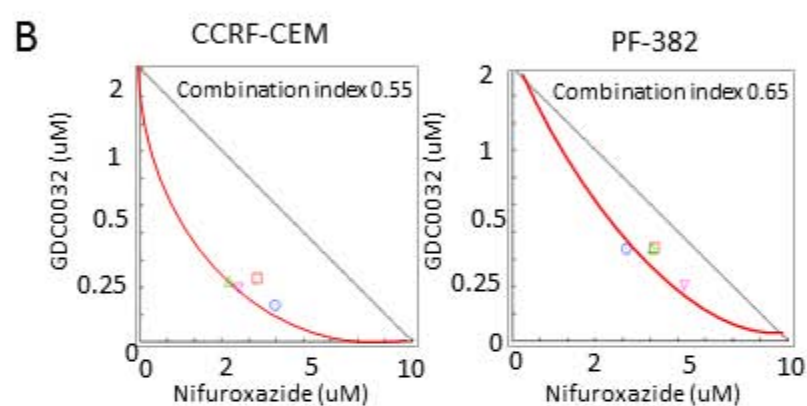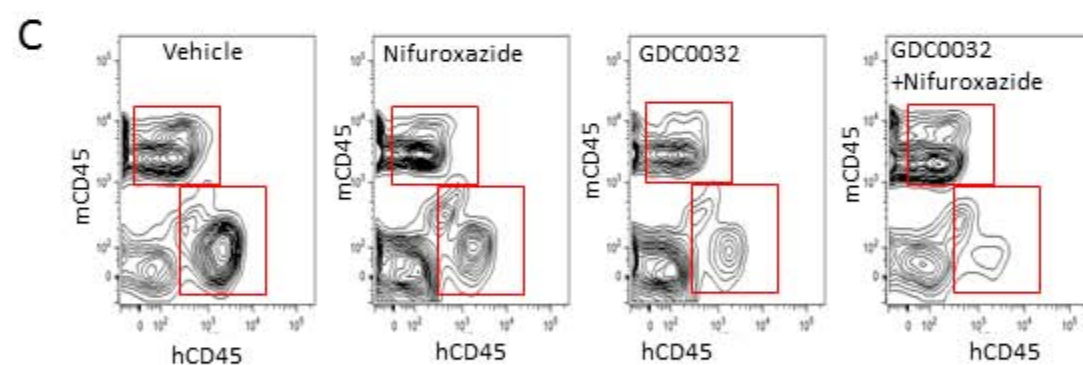

Supplement: Supplementary Figure S2 [file celldisc201630-s2.pdf]
