# Supplementary material for: NTRK2 activation cooperates with PTEN deficiency in T-ALL through activation of both the PI3K–AKT and JAK–STAT3 pathways
Source: Cell Discov. 2016 Sep 20;2:16030–. doi: 10.1038/celldisc.2016.30 (PMC5029543; doi:10.1038/celldisc.2016.30)
Supplement: Supplementary Table S1 [file celldisc201630-s3.pdf]

Supplementary Table.1 Pools of Tel-RTK library and screen hits

Pool-1	Pool-2	Pool-3	Pool-4	Pool-5
pWN-PDGFRB-TEL	pWN-ERBB3-TEL	pWN-DDR1-TEL	pWN-EPHA6-TEL	pWN-EPHB1-TEL
pWN-EPHA3-TEL	pWN-ERBB4-TEL	**pWN-FLT3-TEL**	pWN-FGFR1-TEL	pWN-EPHB6-TEL
**pWN-EPHB4-TEL**	pWN-IGF1R-TEL	pWN-FLT1-TEL	**pWN-FLT4-TEL**	pWN-FGFR3-TEL
pWN-DDR2-TEL	**pWN-NTRK2-TEL**	pWN-MUSK-TEL	pWN-INSRR-TEL	pWN-EGFR-TEL
pWN-NTRK3-TEL	pWN-ZAP70-TEL	pWN-LMTK2-TEL	pWN-TYRO3-TEL	**pWN-ABL1-TEL**
pWN-STYK1-TEL	pWN-MET-TEL	pWN-ALK-TEL	pWN-EPHA2-TEL	pWN-AXL-TEL
pWN-FGFR2-TEL	**pWN-NTRK1-TEL**	pWN-EPHA4-TEL	pWN-ROR2-TEL	pWN-TIE1-TEL
pWN-RET-TEL	pWN-PDGFRA-TEL		pWN-EPHA1-TEL	pWN-KDR-TEL
