# Supplementary material for: NTRK2 activation cooperates with PTEN deficiency in T-ALL through activation of both the PI3K–AKT and JAK–STAT3 pathways
Source: Cell Discov. 2016 Sep 20;2:16030–. doi: 10.1038/celldisc.2016.30 (PMC5029543; doi:10.1038/celldisc.2016.30)
Supplement: Supplementary Information [file celldisc201630-s4.pdf]

Supplementary Figure S1

(A) Immunoblot analysis of PI3K proteins after PTEN knockdown. No change in PI3K regulatory or catalytic isoforms.

(B) The relative growth of each pool (Pools: Poo1, Pool2, Pool3, Pool4 and Pool5) and positive control cells in the presence of IL3. Relative cell growth was calculated compared to shCtrl mock-infected cells (EV). (n=3)

(C) The relative growth of each individual kinase alone in the presence of IL3. Relative cell growth was calculated compared to shCtrl mock-infected cells (EV). (n=3)

(D) Cells expressing NTRK2-Tel or wild-type NTRK2. Comparable expression levels of both NTRK2 were observed.

(E) The relative growth of Ba/F3-shPTEN cells expressing NTRK2-Tel or wild-type NTRK2 in the absence of IL3. Relative cell growth was calculated compared to shCtrl mock-infected cells (EV). (n=3)

Supplementary Figure S2

(A) Phospho-STAT3 levels of PTEN wild-type (TALL-1 and SUPT-11) or mutant (PF382 and CCRF-CEM) T-ALL cell lines.

(B) Isobolograms showing actual (and solid red line) and predicted additive (black line 50% inhibition) effects of combined GDC0032/nifuroxazide therapy on viability of CCRF-CEM and PF382 cells.

(C) Flow cytometry analysis of human CD45+ leukemic cell frequency in Peripheral Blood against mouse CD45 at day 26. PF382-Luc xenografted mice 21 days after initiation of treatment with GDC-0032, or Nifuroxazide or the combination *P < 0.05, **P < 0.01, One-way ANOVA.

Supplementary Table S1. Pools of TEL-RTK library used for the screen and scored kinases.
